# Tuberculosis of Monkey

**Published:** 1896-05

**Authors:** Frank Miller

**Affiliations:** Dog Clinic, Veterinary Academy, Berlin


### ABSTRACTS FROM FOREIGN JOURNALS.

Tuberculosis of Monkey. A two-year-old female monkey, weighing five pounds, was presented in the polyclinic ward for small animals on February 17, 1896, and was admitted to the ward for internal diseases with the following history: "That for three weeks she had eaten but little, and suffered from diarrhœa." After a careful examination the condition of the animal was reported to be as follows:

The patient (giving description) is in a fair state of nutrition; the hair coat is rough and dull; the temperature is properly distributed over the body, and measures 38.2 C. in the rectum; visible mucous membranes are anæmic; appetite is poor; abdomen flaccid, and has "tucked" appearance, and through its walls one can locate a walnut-size nodule of firm consistency in the left lumbar region; the same is comparatively fixed. A somewhat larger body, with less regular contour, is detected immovably situated near the ventral side of lumbar vertebra. The feces are of semifluid consistency, yellowish-brown in color, at times quite fluid. The respiration is 20 to 30 per minute, and of normal type. Percussion of the thorax gives loud, full sound. Auscultation gives a slightly increased vesicular note. The two heart-tones are present. The pulse taken at the arteria femoralis runs from 130 to 150 per minute, and is at times intermittent. Psyche prepossessed. Clinical diagnosis, *tuberculosa intestinalis.*

Upon the two following days, 18th and 19th, there has been no perceptible change in the physical condition beyond a marked reduction of temperature, which was 36.5° and 37.1° C., respectively.

February 20th, patient received 0.01 tuberculin Kochii subcutaneously; temperature reading 36.2° in the morning; 36.3° at 4 P.M.; 36.4° at 8 P.M. February 21st, the temperature averaged 35.4°; February 22d, 34.5°.

Upon the morning of the 22d another injection of 0.01 tuberculin was made. Temperature at 10 A.M., 34.5°; 4 P.M., 34.2°; 6 P.M., 34.1°; 8 P.M., 34.4°.

On the 23d, the temperature averaged 34.5°, and patient showed no change in symptoms beyond slightly increased weakness.

February 24th. The patient lay stretched in cage, regardless of her surroundings. The body is cold; rectal temperature, 34.0°; cardiac impulses imperceptible; respiration extremely shallow. After lying in this condition for several hours was killed by injection of hydrocyanic acid. The records of the case, as found in the Pathological Institute, read as follows:

"*Post-mortem*. Cadaver of female monkey, in fair state of nutrition. Rigor mortis absent. The superficial lymphatic glands are enlarged. Rectal region marked by incrustations of yellowish fecal matter.

"Upon opening the abdominal cavity one sees the mesentery studded with yellowish semi-solid nodules, varying in size from peas to hazelnuts, which, on closer examination, are found to be filled with a truly caseous yellowish-white material. The mesenteric glands are generally enlarged to the size of large beans, and one has the size of a small apple, and contains a fine yellowish substance of mealy consistency. The gastro-intestinal mucous membrane is covered with a thick, slimy layer of mucus, under which may be seen several small points of tissue-change in the submucosa. The spleen is greatly enlarged, and its dark blue-red color is broken by three large walnut-sized nodules of yellowish-white color, one of which on section shows to be composed of a mass of smaller nodules, the other two are broken down and filled with material similar to that noted in the mesenteric nodules. There are many small nodules scattered through the splenic parenchyma.

"The kidneys are likewise studded with innumerable small yellowish nodules, situated both on Glisson's capsule and in the parenchymatous substance of the organ, and particularly in the corticular layer. The organs are changed to a dirty gray-brown color, with roughened surface.

"The genital system, including uterus, Fallopian tubes, and ovaries, are almost unrecognizably changed by the deposits of nodules.

"The peritoneum shows upon the left side a few caseated nodules of small size. The pleura costalis of the right side shows similar change.

"The left ventricle of heart shows a yellowish-white nodule of pin's-head size in its muscular wall.

"The lungs in a few localized areas are more firm in substance, especially on left diaphragmatic lobe, and are yellowish-gray in appearance. In this part one can feel several small firm nodules. The bronchial glands are enlarged and changed to caseated masses. Bacteriological examination of nodules revealed multitudes of tubercle bacilli.

"Pathological diagnosis, *tuberculosa generale*."

The early collapse of this case can be in a great measure accounted for by the administration of the tuberculin, which has in many cases shown the presence of the disease by a marked diminution of temperature instead of rise, especially in the monkey family.—FRANK MILLER, V. S., *Dog Clinic*, *Veterinary Academy*, *Berlin*.

